# Ahmed implant coated with poly(2-methacryloyloxyethyl phosphorylcholine) inhibits foreign body reactions in rabbit eyes

**DOI:** 10.1371/journal.pone.0252467

**Published:** 2021-05-28

**Authors:** Hyun Joo Kee, Eun Jung Lee, Jong Chul Han, Changwon Kee

**Affiliations:** Department of Ophthalmology, Samsung Medical Center, Sungkyunkwan University School of Medicine, Seoul, Korea; University College London Institute of Child Health, UNITED KINGDOM

## Abstract

**Purpose:**

Wound healing after Ahmed glaucoma valve (AGV) implantation often entails fibrosis as a foreign body reaction to the silicone plate. Poly(2-methacryloyloxyethyl phosphorylcholine) (PMPC) forms an antifouling surface that inhibits fibrosis during wound healing. In this study, we aimed to compare the effects of the implantation of AGV coated with PMPC (wPMPC) versus AGV without PMPC (woPMPC) in rabbits.

**Methods:**

Six New Zealand White rabbit does underwent AGV implantation in both eyes. For each rabbit, one eye was randomly selected for implantation of AGV wPMPC and a conventional AGV (woPMPC) was implanted in the contralateral eye. Gross conjunctival vascularity was compared between the two groups at the first, second, and fourth weeks after surgery. The eyes were enucleated in four weeks and subjected to staining with hematoxylin and eosin and Masson’s trichrome stain. The fibrosis and inflammation status among the eye samples were compared by measuring the thickness of the fibrotic walls and counting the number of chronic inflammatory cells around the AGV. Counting of inflammatory cells and measuring fibrotic wall thickness were done in a blinded method to eliminate observer bias. Statistical analysis was performed using the Mann-Whitney U test.

**Results:**

Gross and histological examinations revealed no toxic effects of PMPC. There were no apparent differences in overall conjunctival vascularity between the two groups at weeks 1, 2, and 4 after surgery. The average inflammatory cell counts were 14.3 ± 5.8 per slide and 27.3 ± 8.6 per slide in the wPMPC and woPMPC groups, respectively (*p* = 0.037). The average thicknesses of the fibrotic wall were 57.9 ± 11.3 μm and 81.5 ± 21.3 μm in the wPMPC and woPMPC groups, respectively (*p* = 0.025).

**Conclusion:**

Compared to the woPMPC group, the number of inflammatory cells and fibrosis were significantly decreased in the wPMPC group.

## Introduction

The healing response associated with glaucoma surgery is highly variable and a major determinant of long-term surgical outcomes [1, 2]. Excessive fibrosis is one of the major reasons for unsuccessful outcomes in filtration surgery [1, 3, 4]. Few agents are used to suppress fibrosis, including anti-inflammatory drugs (e.g. corticosteroids) or antimetabolites (e.g., mitomycin C (MMC) and 5-fluorouracil). However, even when these agents are administered intraoperatively and postoperatively, fibrosis may occur and lead to irreversible bleb failure [5, 6]. Likewise, excessive fibrosis surrounding the plate of the glaucoma implant may result in unfavorable outcomes in drainage implant surgery [7]. The effectiveness of MMC treatment in drainage implant surgery in glaucoma has been evaluated in certain studies. A retrospective study found the procedure to be effective [8], whereas in other randomized controlled trials, there was no evidence that treatment with MMC helped improve the success rate [9, 10].

2-Methacryloyloxyethyl phosphorylcholine (MPC) has a side chain that consists of anionic phosphate and cationic quaternary ammonium in close proximity, producing a zwitterionic repeat unit in the polymer. Poly(2-methacryloyloxyethyl phosphorylcholine) (PMPC) has phosphorylcholine head groups similar to those present in the outer leaflet of lipid bilayers such as phosphatidylcholine and sphingomyelin in mammalian membranes [11]. Therefore, PMPC mimics the outer membrane surface chemistry and acts as a shield to prevent significant cell adhesion and tissue reactions. PMPC inhibits protein adsorption and cell adhesion to surfaces when in contact with plasma or whole blood. These characteristics are attributed to the extreme hydrophilicity and electrically neutral nature of the polymers, as well as to the ability of phosphorylcholine to combine with surrounding water and form a firm hydration shell [12, 13]. This property of PMPC enables its medical use in long-term implants [14]. Several PMPC-based high performance artificial organs have been introduced in literature. To improve the blood compatibility of a small-diameter vascular prosthesis, prostheses containing segmented polyurethane and PMPC were made and grafted to carotid arteries of rabbits, and the prostheses were patent without thrombus and pseudointima [15]. Chen et al. [16] found that a PMPC-grafted stainless steel composite showed better osteoblast biocompatibility and antibacterial effect against *staphylococcus aureus* than a pristine stainless steel surface. Park et al. [17] performed an in vivo study with PMPC-coated silicone breast implants in rats and observed a reduction of nonspecific protein adsorption and fibroblast adhesion on the implant surface. Furthermore, they found a significant decrease in inflammation-related cells, transforming growth factor (TGF)-β and myeloperoxidase level compared to non-coated implants. An α-smooth muscle actin and collagen density around the PMPC-coated implants were also significantly decreased compared to non-coated implants.

The Ahmed glaucoma valve (AGV) currently in use is made of silicone elastomer. Therefore, its surface is hydrophobic. Coating of the implant with PMPC renders the surface hydrophilic [17]. The hydrophilic surface does not trigger adhesion to, and proliferation of, inflammatory cells at high levels [18]. Moreover, the hydrophilic nature reduces the adsorption of proteins on the surface, which reduces the intensity of foreign body reactions [12]. Based on these findings, we attempted to coat a drainage implant with PMPC and suppress fibrosis following glaucoma implant surgery. Several studies have proposed different modifications to glaucoma drainage devices. The novel devices include an adjustable glaucoma device [19], a device delivering cyclosporine A [20] or MMC [21] for a certain period, and a device that contains a plate made of polymethyl methacrylate [22], or a plate replaced with microfluidic meshwork [23]. To our knowledge, this is the first preliminary study to demonstrate the use of a novel coating material for fibrosis inhibition in glaucoma implant surgery. The purpose of this study was to evaluate the effects of PMPC-coated Ahmed glaucoma implants in a rabbit model.

## Materials and methods

### Animals

Six New Zealand White rabbit does weighing between 2.1 and 2.5 kg were purchased from Dooyeoul Biotech, Inc (Seoul, Korea). The Institutional Animal Care and Use Committee (IACUC) of the Samsung Biomedical Research Institute approved the study (Permit number: 20181228002). All animals were treated in compliance with the Association for Research in Vision and Ophthalmology Statement for the Use of Animals in Ophthalmic and Vision Research. All of the rabbits took an acclimatization period for 1 week prior to initiation of the study. Rabbits were individually housed with food and water. The cages were set at a 12-hour light-dark cycle, temperature of 18–22°C and 30~70% relative humidity. Commercial rabbit feed (Purina® rabbit feed; Cargill Agri Purina, Inc, Gyeonggi-do, Korea) in the form of pellets was delivered once a day (50g/kg) while the drinking water was provided ad libitum. Cages and feeder racks were cleaned daily before they were fed. All rabbits were inspected daily for abnormal behavior or defecation. They were weighed daily for the first week and then weighed weekly thereafter. Topical and general anesthesia using a proparacaine eyedrop and tiletamine with zolazepam mixture were done in study procedures when needed, as approved in the IACUC protocol. All animals were humanely euthanized after general anesthesia using appropriate euthanasia agents (potassium chloride).

### Study design

Six rabbits were subjected to conventional glaucoma valve implantation surgery in one eye with an uncoated Ahmed valve (woPMPC) (Model FP8 Ahmed Glaucoma Valve; New World Medical, Inc., Rancho Cucamonga, CA). The rabbits were also subjected to the same surgery in the other eye with an AGV coated with PMPC (wPMPC). The eye laterality was randomly selected by simple randomization.

### Production of PMPC-coated AGV implants

**Fig 1:**
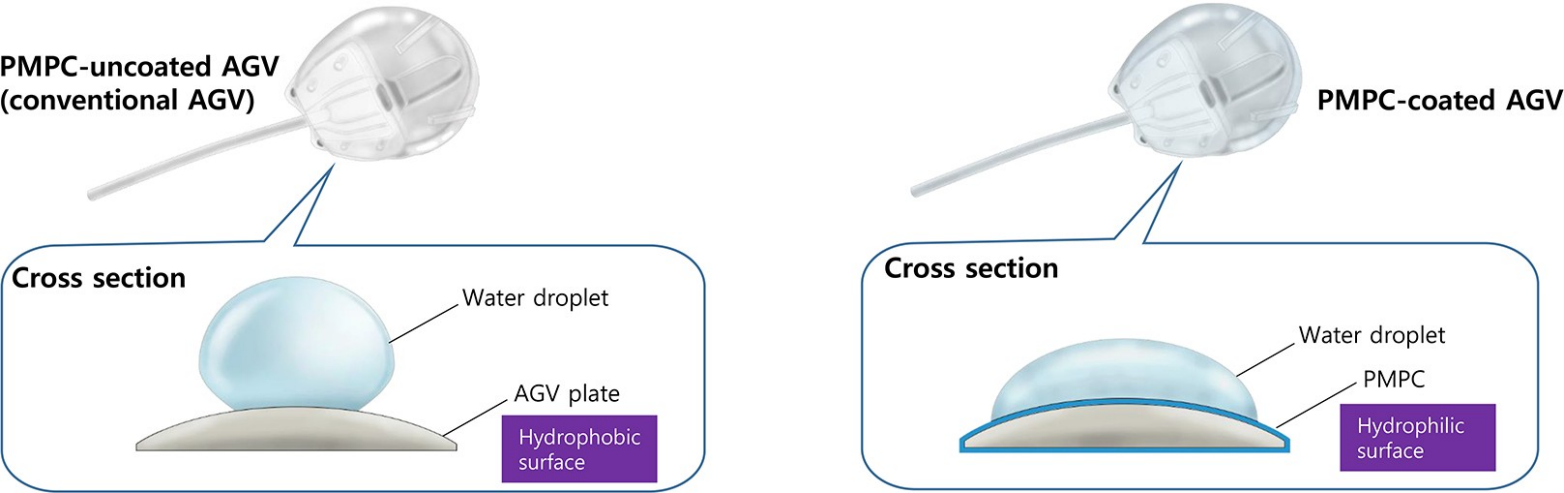
Comparison of the surface nature of PMPC-uncoated Ahmed glaucoma valve (AGV) and PMPC-coated AGV. The water contact angle was over 90° for the conventional AGV; therefore, the AGV originally had a hydrophobic surface. When the conventional AGV was coated with PMPC, the water contact angle decreased to below 90°, which implied that PMPC-coated AGVs have a hydrophilic surface.

The AGV was submerged for a minute in an initiator solution containing benzophenone (55 mM) and dipentaerythritol penta-/hexa-acrylate (2.5 mM) in acetone followed by vacuum drying for an hour. The benzophenone-adsorbed AGV was immersed in monomer solution containing an MPC monomer (0.25 M) and ethylene glycol dimethacrylate (5.0 mM) in deionized water. Then the AGV was treated with ultraviolet irradiation for 15 minutes using a 600 W high-pressure mercury lamp (MS Tech, Gyeonggi-do, Korea). By thoroughly rinsing with acetone and water, unreacted monomers, benzopinacol, and excess benzophenone were removed. Finally, any remaining acetone and non-covalently bound polymers were removed by soaking the coated AGV in water overnight [17]. The process was conducted at the chemistry department at the Seoul National University. Six AGVs wPMPC were prepared using this procedure. The AGVs woPMPC had hydrophobic surfaces, whereas the AGVs wPMPC had hydrophilic surfaces. This was confirmed using contact-angle measurements (Fig 1).

### Surgical procedure

All operations were performed by the same surgeon (JCH). For general anesthesia, 15 mg/kg tiletamine with zolazepam mixture (Zoletil^®^ 50; Virbac, Carros, France) was injected intramuscularly. Proparacaine hydrochloride (Paracaine^®^ 0.5%; Hanmi Pharmaceutical, Seoul, Korea) was applied topically to each eye.

Normal saline (0.9% NaCl) was administered via subtenon injection to separate the Tenon’s capsule from the outer surface of the sclera. A fornix-based flap of the conjunctiva and Tenon’s capsule in the superotemporal quadrant was raised, and posterior dissection was performed using Westcott tenotomy scissors. Priming was accomplished by injecting 1 ml of normal saline through the drainage tube using a blunt 27-gauge cannula. The plate was placed inside the pocket of the conjunctiva and Tenon’s capsule and secured using 9–0 nylon sutures (Ethilon^®^; Ethicon, Inc., Somerville, NJ, USA) at 3 mm behind the limbus. The tube tip was cut obliquely to permit 2 mm insertion of the tube into the anterior chamber. A tract was formed from 1.5 mm posterior to the limbus by advancing a 23-gauge needle toward the anterior chamber. Sodium hyaluronate (1.65%) and sodium chondroitin sulfate (4%) viscoelastic (DisCoVisc^®^; Alcon Laboratories, Inc., Geneva, Switzerland) was injected into the anterior chamber, and the tube was inserted through the tract. The tube was then loosely secured to the sclera using a 9–0 nylon suture. The conjunctiva was sutured using 8–0 polyglactin sutures (Vicryl^®^; Ethicon, Inc.). After the procedure, 3 mg/g ofloxacin ophthalmic ointment (Tarivid^®^; Santen Pharmaceutical, Osaka, Japan) was applied to each eye.

The images of gross vascularity of the conjunctiva were acquired at weeks 1, 2, and 4. To obtain a good impression of the whole superotemporal bulbar region, the eyes were pulled towards the medial side with forceps under topical anesthesia. All rabbits were sacrificed for histopathological examination four weeks after the surgery. A 15 mg/kg tiletamine-zolazepam mixture (Zoletil^®^ 50; Virbac, Carros, France) was injected intramuscularly for anesthetization, and the animals were euthanized by injecting 1–2 mmol/kg of potassium chloride intravenously. The eyes were then enucleated for pathological examination.

### Gross conjunctival vascularity

After the surgery, the images of each eye were acquired at weeks 1, 2, and 4 before euthanasia. The overall severity of hyperemia in the superotemporal region of each eye was graded by another author (HJK). The Indiana Bleb Appearance Grading Scale was used since it is the most widely adopted scale and provides clinical judgment for the objective measurement of bulbar redness [24]. The grades are provided on a scale of 0 to 4, with a score of 4 representing samples with the highest vascularity.

### Histology

The extracted eyeballs were immersed in 4% paraformaldehyde prepared in 0.1M phosphate-buffered saline (PBS) for three hours. The eyeballs were sectioned in the sagittal plane along the midline of the implant using a razor blade and rinsed with 0.1 M PBS. The samples were then processed and embedded in embedding medium (polyethylene glycol 400 distearate; Polysciences, Inc., Warrington, PA, USA). The blocks were sectioned in the sagittal direction at a thickness of 5 μm using a microtome, and the resulting sections were placed on slides. The 5-μm-thick tissue sections were rehydrated and stained with hematoxylin and eosin (H&E) and analyzed to evaluate the overall inflammatory changes in the tissues. The number of inflammatory cells was counted in the entire tissue surrounding the implant. The total number of chronic inflammatory cells, including macrophages, monocytes, lymphocytes, plasma cells, and foreign body giant cells were counted. The collagen deposits were assessed by Masson’s trichrome staining analysis. The fibrous capsule thickness was measured as the average of the minimum and maximum thicknesses of each six random fields among the total fibrous capsule. The measurements of inflammatory cell counts and fibrous capsule thickness were done in a blinded method. Numerical data were obtained using a pathology slide viewing software (Aperio ImageScope, version 12.1; Leica Biosystems Imaging, Inc., Buffalo Grove, IL, USA).

### Inflammatory cell count

**Fig 2:**
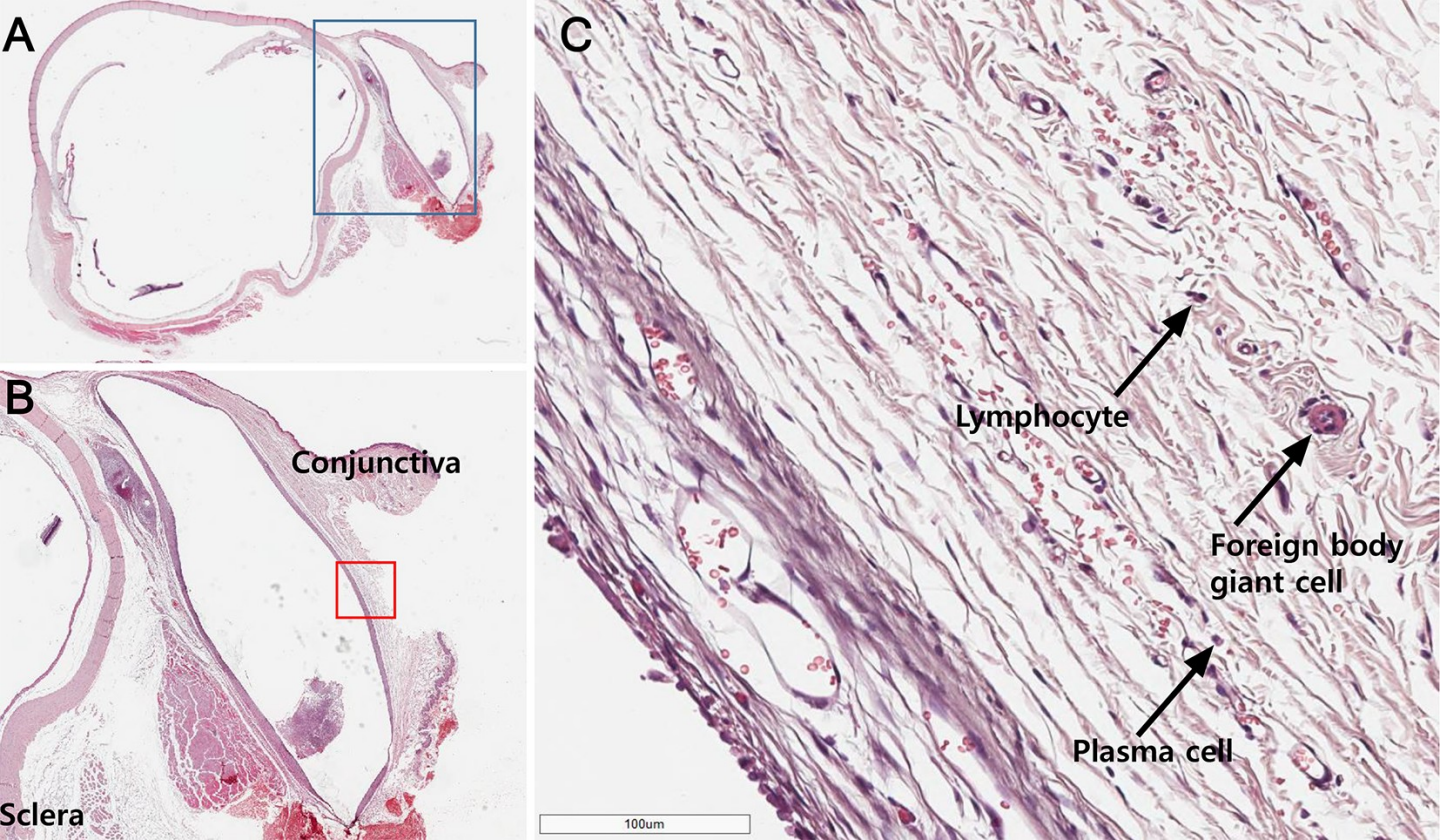
Methods for measuring the average inflammatory cell count. (A) Hematoxylin and eosin staining of a microsection crossing the midline of the implant. The blue box indicates the surgical site. (B) 8× magnified image of the blue box in Fig A. The number of inflammatory cells in the tissue surrounding the implant was counted, following which the average number of cell counts of each group was calculated. (C) 200× magnified image of a red box marked in Fig B. Various inflammatory cells such as lymphocytes, foreign body giant cell, plasma cells are seen.

A 5-μm section was stained with H&E to count the numbers of chronic inflammatory cells. The measured location was confined to the tissue surrounding the implant area. The number of inflammatory cells in each slide was counted, and the average number of inflammatory cells in six slides was calculated for each group (Fig 2).

### Fibrotic wall thickness

**Fig 3:**
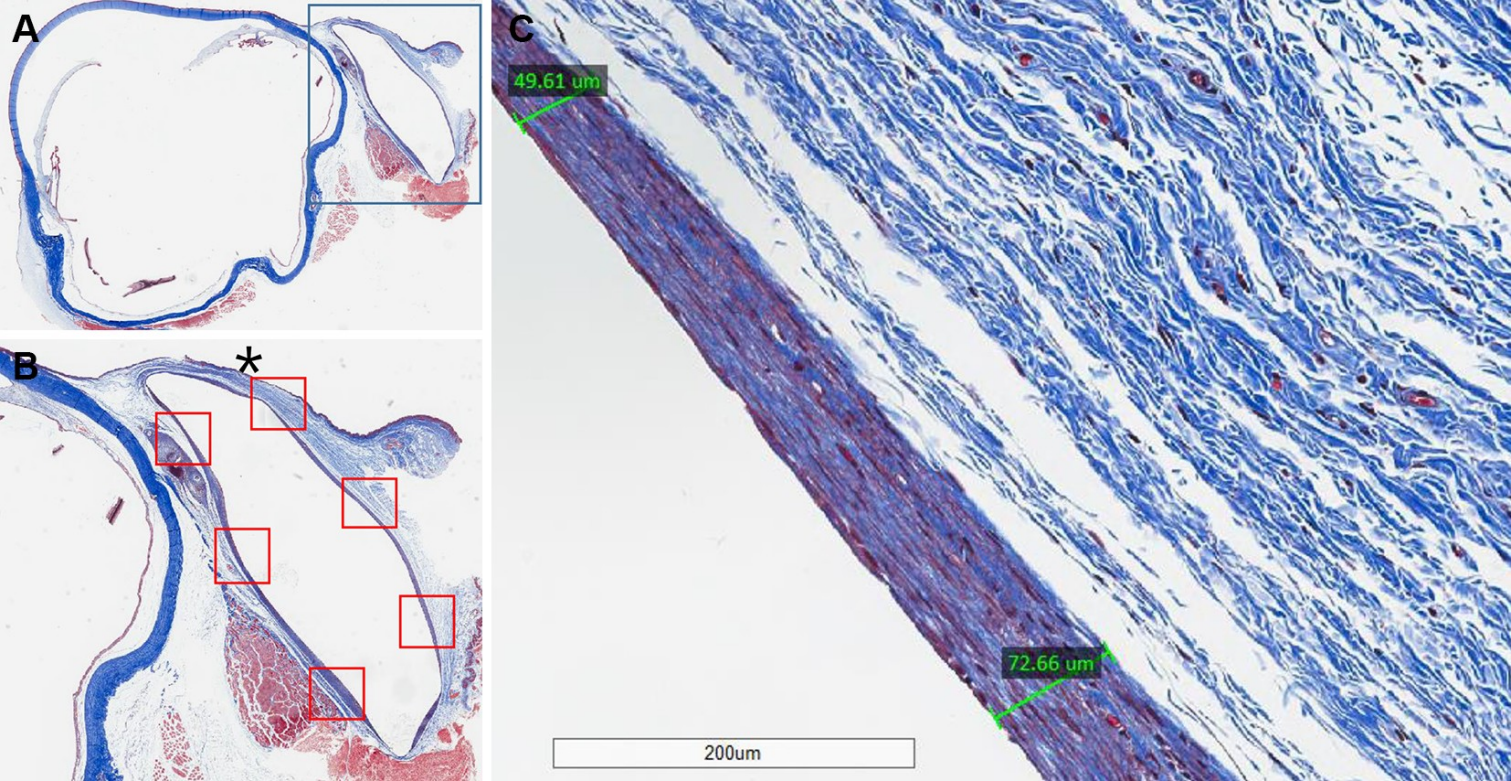
Methods for measuring average fibrotic wall thickness. (A) Masson’s trichrome staining of a microsection across the midline of the implant. The blue box indicates the surgical site. (B) 8× magnified image of the blue box in Fig A. Six parts of fibrotic capsule around the implant were selected. (C) 125× magnified image of a red box marked with the asterisk in Fig B. The fibrotic wall thickness was measured by averaging the length of the thinnest and thickest lines. The final mean data were collected by averaging the values corresponding to each of the six parts (six red boxes in Fig B).

The thickness of the innermost avascular collagenous layer was measured by Masson’s trichrome staining analysis. The fibrotic wall thickness varied slightly depending on the location. Six different parts of the fibrotic wall surrounding the implant were measured (Fig 3B). The average thickness was calculated by measuring the lengths of the thinnest and thickest sections in each of the six parts, and the six values were then averaged (Fig 3C).

### Statistical analysis

Values are presented as the means ± standard deviations. Statistical analyses were performed using SPSS statistical software (version 24.0; SPSS, Inc., Chicago, IL, USA). The differences in the number of inflammatory cells and thickness of the fibrotic wall between the wPMPC and woPMPC groups were analyzed using the Mann-Whitney U test. Differences with P values < 0.05 were deemed to be statistically significant.

## Results

We did not observe any serious ocular complications such as endophthalmitis, choroidal detachment, or implant exposure during the intraoperative and 4-week postoperative period.

### Gross conjunctival vascularity

**Fig 4:**
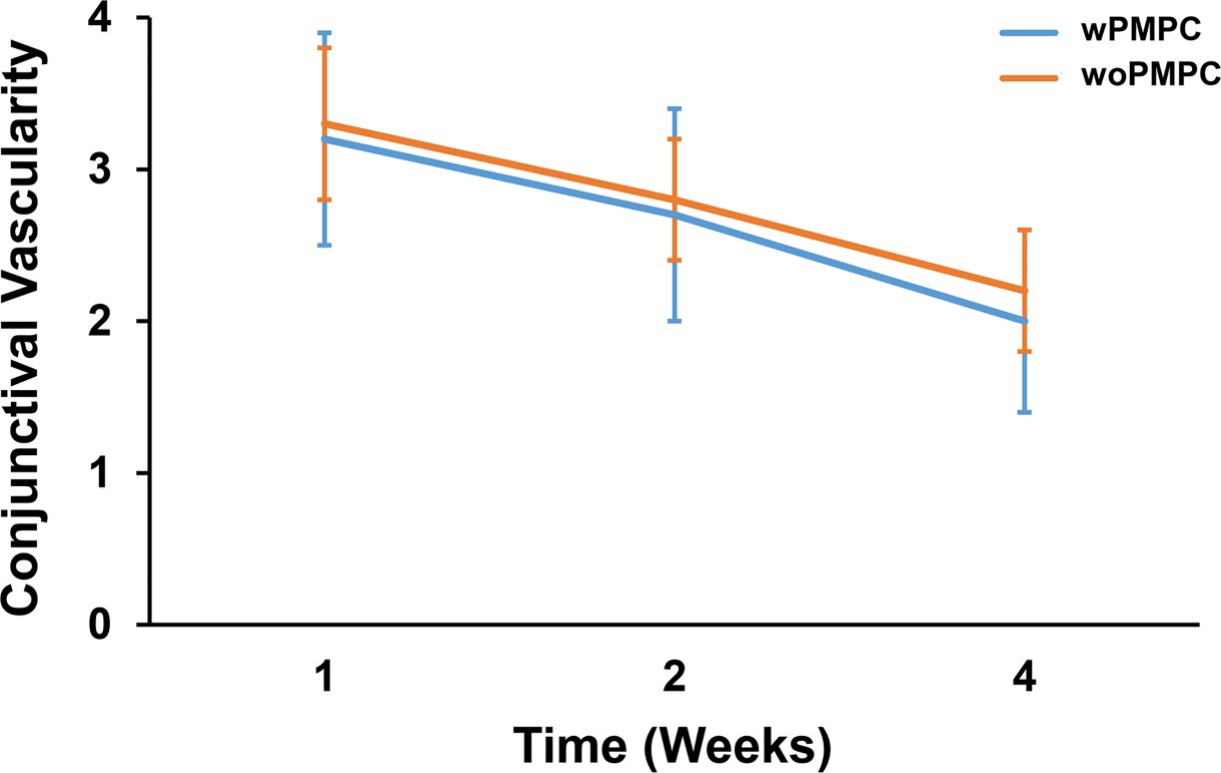
Comparison of gross conjunctival vascularity. Quantification of overall conjunctival vascularity using the Indiana Bleb Appearance Grading Scale. There were no significant differences between the two groups at weeks 1, 2, and 4 after surgery (p = 0.715, 0.523, 0.598, respectively). Data are shown as the mean ± standard deviation for each group. wPMPC, Ahmed glaucoma valve coated with PMPC; woPMPC, Ahmed glaucoma valve without PMPC-coating.

The comparison of conjunctival vascularity between the AGV wPMPC-implanted eye and AGV woPMPC-implanted eye of a single rabbit during the 4-week postoperative period is presented in S1 Fig. Animals from both the wPMPC and woPMPC groups exhibited moderate hyperemia within 1 week after surgery. However, the injection decreased 2 weeks after the surgery in both groups. There were no apparent differences between the eyes from the two groups at 4 weeks (Fig 4).

### Inflammatory cell count

**Fig 5:**
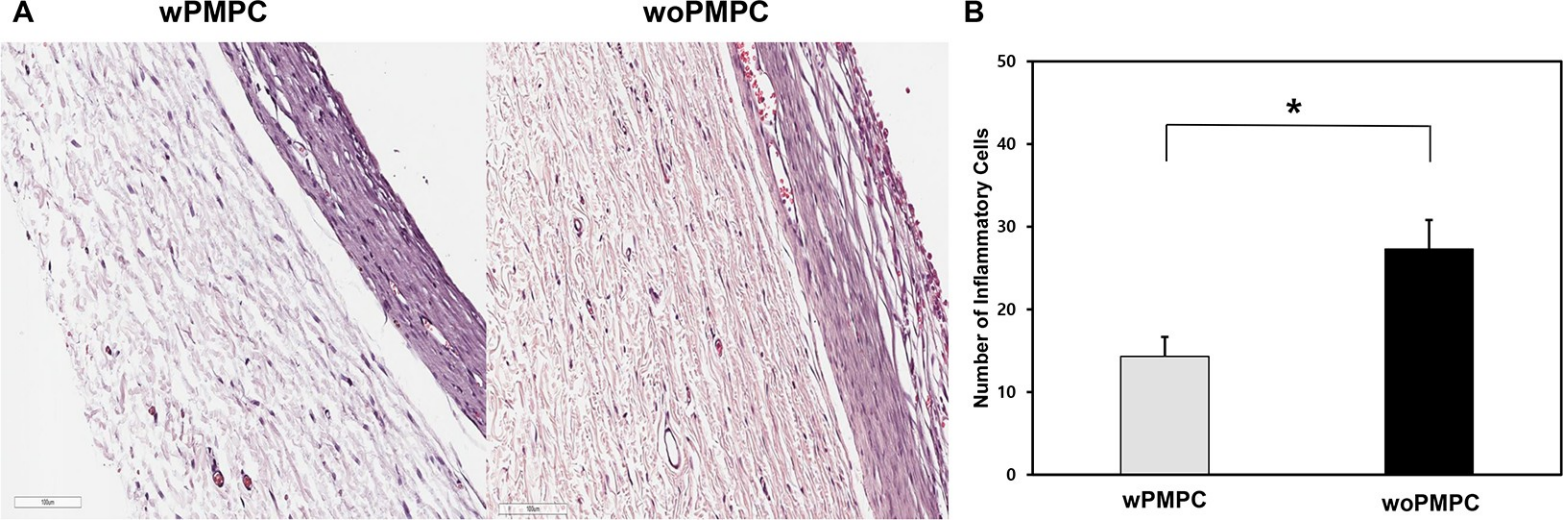
Comparison of inflammatory response in tissues surrounding the implant area after Ahmed glaucoma valve implantation. (A) 200**×** magnified images of H&E staining showed numerous inflammatory cells of each eye in the same rabbit. Scale bars: 100 μm. (B) The number of inflammatory cells around the PMPC-coated AGV was significantly smaller than that around the PMPC-uncoated AGV (*p* = 0.037). Data are shown as the mean ± standard deviation for each group. wPMPC, Ahmed glaucoma valve coated with PMPC; woPMPC, Ahmed glaucoma valve without PMPC-coating.

The average number of inflammatory cells was determined by histological analysis of the H&E-stained sections of the specimen (Fig 5A). The eyes that underwent glaucoma drainage implant surgery with the AGV wPMPC were compared with control eyes that underwent surgery with the AGV woPMPC (Fig 5B). The average number of inflammatory cells per slide in the wPMPC group was 14.3 ± 5.8 compared to 27.3 ± 8.6 in the woPMPC group, a difference that was statistically significant (*p* = 0.037).

### Fibrotic wall thickness

**Fig 6:**
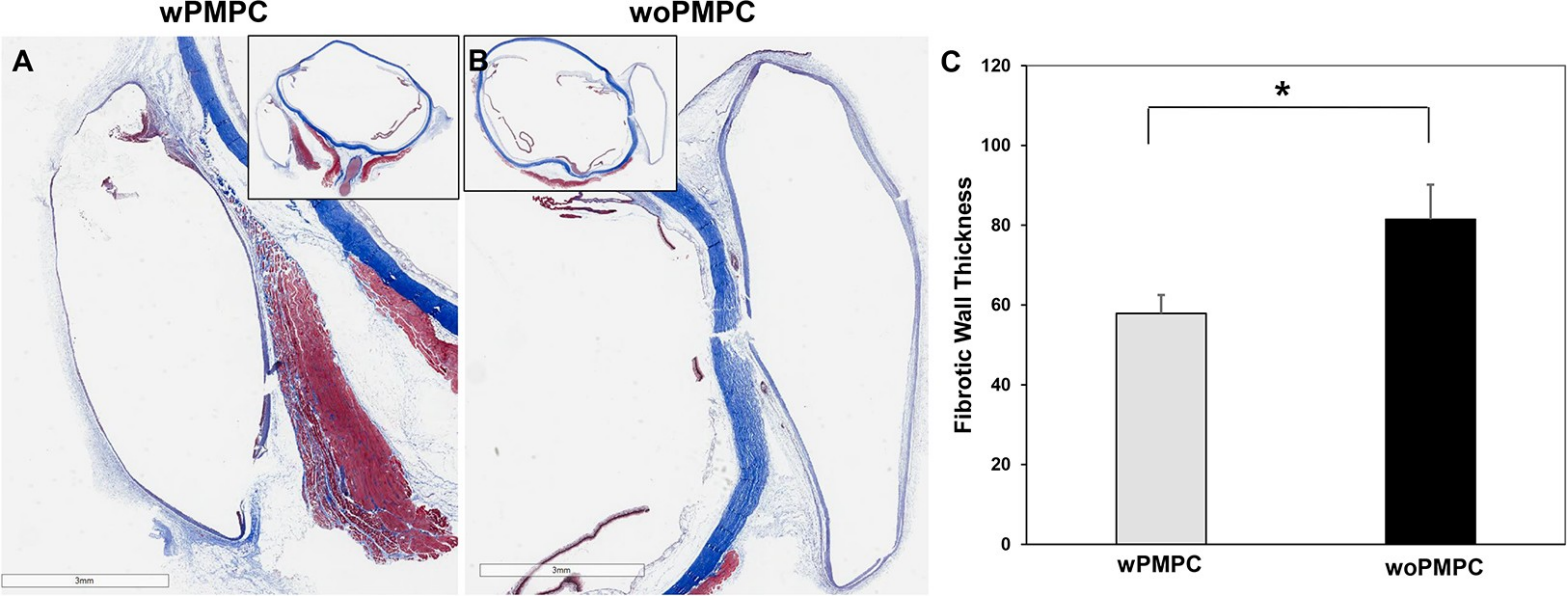
Comparison of overall thickness of the fibrotic wall. (A) The main image shows 8**×** magnified image of the fibrotic wall region in the thumbnail image. The fibrotic capsule around the PMPC-coated implant is stained modest blue. (B) The fellow eye of the same rabbit with PMPC-uncoated implant. Scale bars: 3 mm. (C) In the wPMPC group, the average fibrotic wall thickness was significantly thinner than that in the woPMPC group (p = 0.025). Data are shown as the mean ± standard deviation for each group. wPMPC, Ahmed glaucoma valve coated with PMPC; woPMPC, Ahmed glaucoma valve without PMPC-coating.

Masson’s trichrome stained the fibrotic wall modestly blue, which made easy to distinguish it from sclera and Tenon’s tissue (Fig 6A and 6B). The average thicknesses of the fibrotic wall in the eyes implanted with the AGV wPMPC and woPMPC were 57.9 ± 11.3 μm and 81.5 ± 21.3 μm, respectively (*p* = 0.025) (Fig 6C).

## Discussion

This study demonstrated the efficacy of PMPC-coated Ahmed glaucoma implants in a rabbit model. The average inflammatory cell count and average thickness of the fibrotic wall were significantly lower when PMPC-coated implants were used than when non-coated implants were used. In this study, the PMPC-coated Ahmed glaucoma implants suppressed foreign body reactions, compared to the case for the controls.

The success rate of glaucoma valve implantation was reported to be 82.7%–93.9% at first year postoperatively, whereas it decreased to 65.0%–70.2% five years later [25–27]. During a five-year follow-up, encapsulation of the bleb around the plate of the Ahmed glaucoma implant accounted for 0.9%–11% of the complications encountered [28, 29]. Therefore, an additional attempt to reduce bleb scarring is necessary.

There have been several pharmaceutical approaches to regulate wound healing, such as controlling the levels of TGF-β using 5-fluorouracil and MMC [30]. However, these agents are non-selective and have been reported to induce severe damage by causing complications such as hypotony, corneal endothelial loss, and bleb-related endophthalmitis causing blindness [31–33]. Surgical interventions to reduce conjunctival scarring include the use of collagen-glycosaminoglycan implant, fibrin glue, and amniotic membrane transplantation [34]. A therapy for preventing conjunctival contraction and scarring during the wound healing process is often necessary.

The normal wound healing response in the conjunctiva is divided into four phases: hemostasis, inflammation, proliferation, and remodeling. PMPC seems to inhibit the hemostatic phase. When silicone implant is implanted, rapid activation of the host immune system in response to silicone material triggers a foreign body reaction [35]. Within a few minutes, host proteins from nearby tissues and blood adhere to the silicone surface, and the adsorbed proteins form a layer with a modified three-dimensional structure [36]. Proteins such as fibrin, components of the complement system, and interleukins attract macrophages by releasing proinflammatory signals. As the implant has a considerably larger surface area than a macrophage, the accumulated macrophages fuse and form foreign body giant cells on the biomaterial surface. These inflammatory cells secrete mediators stimulating fibroblast migration, and a fibrous capsule is formed around the implant [37]. Protein adsorption is the first phenomenon that occurs when a synthetic material is implanted in a living organism. The uncontrolled protein adsorption triggers foreign body reaction from a host. Thus, nonfouling nature of PMPC reduces foreign body reaction [14]. A fibrous capsule surrounding a synthetic material results from a foreign body reaction [37]. A capsule formation can be curbed by interfering with initiating events–nonspecific protein adsorption–in the foreign body reactions [38]. Therefore, this implies that PMPC can suppress fibrosis. Most proteins in human body fluids generally harbor a weak net negative charge at pH 7 [39]. When a protein adheres to surface and loses bound water at the surface-contacting proportion, protein denaturation occurs. Ishihara [40] reported that proteins adsorbed on the surface of PMPC could keep their original α-helix level higher than that on poly(2-hydroxyethyl methacrylate). Therefore, various chemical strategies for grafting biomaterials that possess electrically neutral or weakly negative surfaces and also have good surface wettability have been studied [41, 42]. Zwaal et al. [43] reported that the inner membrane of red blood cells caused a thrombogenic response, whereas the outer membrane did not. The suggested theory was that the lipid components constituting the outside surface of the red blood cell membrane are mainly zwitterionic phospholipids (such as phosphatidylcholine), whereas the inner components are negatively charged (such as phosphatidylserine). Inspired by the natural cell membrane lipid research, PMPC has been subsequently developed [11].

Some studies have reported the results of implanting polymer-coated implants in primates. Bjugstad et al. [44] investigated the biocompatibility of polyethylene glycol-based hydrogels in the non-human primate brain and showed the absence of T cells and gliotic scarring in any implanted hemisphere. Safley et al. [45] examined the function of microencapsulated adult porcine islets transplanted in streptozotocin-diabetic non-human primates. The adult porcine islets were encapsulated in alginate. Although the grafts eventually failed in all recipients, the explanted microcapsules were intact without evidence of fibrosis and some still reduced hyperglycemia when re-implanted in diabetic mice. Moreover, phosphorylcholine polymer-based drug-eluting stents have been applied to patients with carotid artery disease and had lower rates of myocardial infarction than bare-metal stents and showed long-term safety [46].

In previous studies, the use of PMPC-coated hydrophilic surfaces dramatically reduced the adsorption of proteins and fibrinogens [17, 40]. Moreover, clinical applications of PMPC in silicone implants showed that they exhibit high biocompatibility as they induce lower levels of fibrous capsular formation and also inhibit the expression of inflammatory markers, such as myeloperoxidase, TGF-β, and α-smooth muscle actin, in surrounding tissues by 20%–30% compared to non-grafted implants [17, 47]. The present study also showed that the AGVs wPMPC suppress fibrosis in the conjunctiva by reducing the number of inflammatory cells and the fibrous capsular thickness.

We used pediatric FP8 AGV instead of adult FP7 AGV since the mean axial length of an eye in New Zealand White rabbits is 15.1 mm [48], which is considerably shorter than that of human adults. However, the outcomes of FP 8 versus FP7 AGV in adult secondary glaucoma came out to be comparable in a previous study [49]. Therefore, the usage of FP8 AGV does not appear to be a major determinant influencing the results.

PMPC‐based biomaterials have been used for approximately 40 years, and several medical devices containing PMPC have received FDA approval with regard to the safety aspect [14]. We did not observe any harmful effects of AGVs wPMPC in rabbit eyes; however, further studies for assessing cell viability, chromatin condensation, and free radical production may be conducted before the application of these implants to humans [50].

This study had several limitations. First, the number of rabbits used in this study was relatively small. Nevertheless, we assume that even a small population is enough for a study for introducing a novel material, such as the previous study using three rabbits to test the biocompatibility of microfluidic meshwork in Ahmed glaucoma valve surgery [23]. Second, this was a short-term study involving a 4-week observation period. The wound healing response in rabbits has been reported to be abrupt compared to that in humans; glaucoma filtration surgeries in rabbits fail within 2–3 weeks postoperatively owing to aggressive wound healing at the surgical site [51]. With respect to the glaucoma drainage device implantation, fibrous encapsulation was formed over the first 3–6 weeks after Baerveldt drainage device implantation [52]. Another major drawback of this study is that we did not measure the intraocular pressures. However, we believe that the measurement of the intraocular pressure is unimportant unless we use a glaucoma model in rabbits, as AGV operates in the hypertensive range.

In conclusion, the AGV wPMPC significantly suppressed inflammation and fibrosis after its implantation in rabbit eyes. The implantation of PMPC-coated glaucoma valves in the future may increase the success rate of ocular implant-based surgeries.

## Supporting information

S1 Fig Representative cases comparing gross conjunctival vascularity. Images showing the longitudinal changes in conjunctival vascularity encompassing the implant site. The eyes shown in the left column were implanted with Ahmed glaucoma valve (AGV) coated with PMPC. The right column shows the fellow eye of the same rabbit implanted with AGV without PMPC-coating.
(TIF)

S1 Data (XLSX)
