# Supplementary figures and images for: Ahmed implant coated with poly(2-methacryloyloxyethyl phosphorylcholine) inhibits foreign body reactions in rabbit eyes
Source: PLoS One. 2021 May 28;16(5):e0252467. doi: 10.1371/journal.pone.0252467 (PMC8162657; doi:10.1371/journal.pone.0252467)

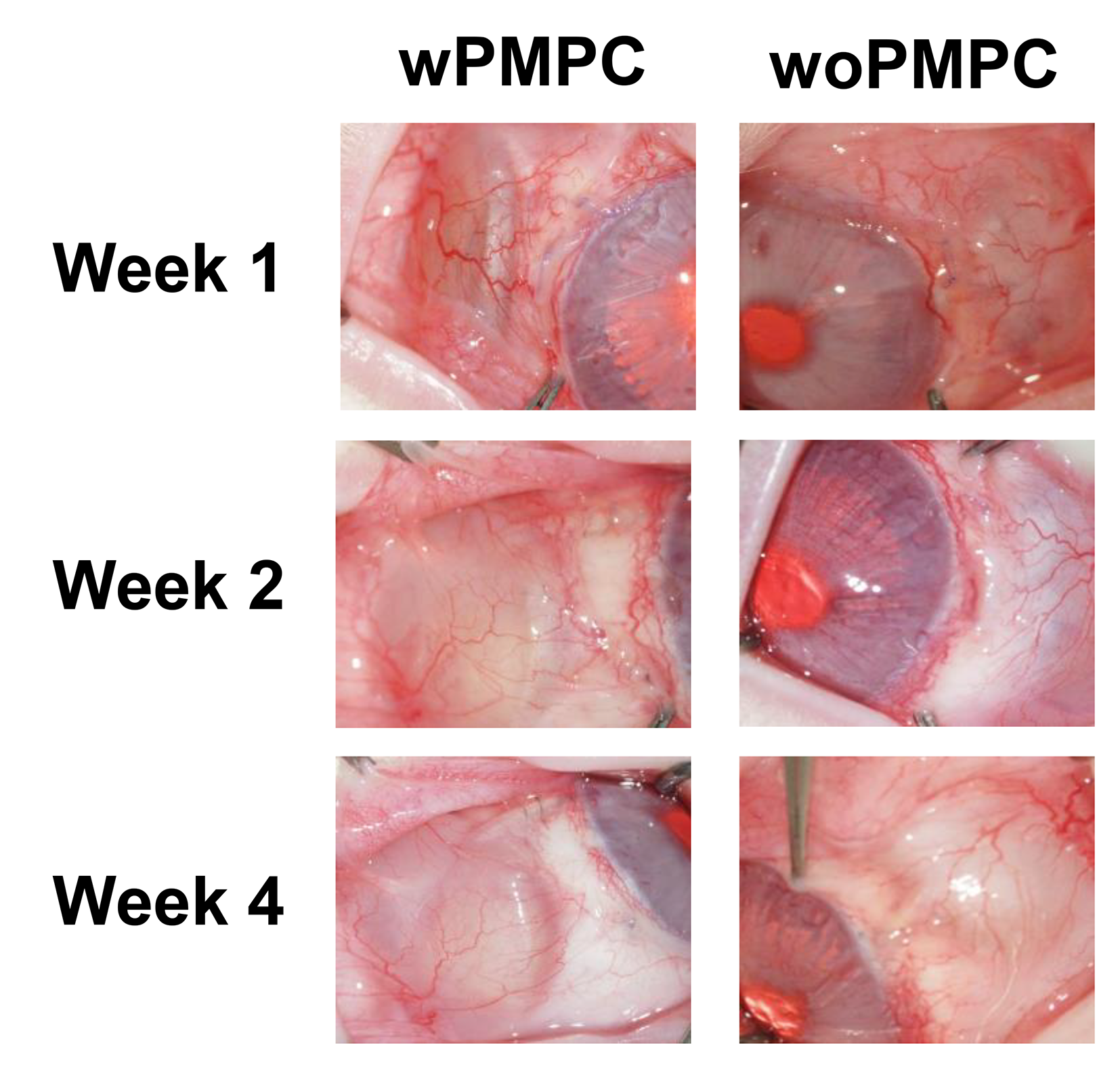

Supplement: S1 Fig — Images showing the longitudinal changes in conjunctival vascularity encompassing the implant site. The eyes shown in the left column were implanted with Ahmed glaucoma valve (AGV) coated with PMPC. The right column shows the fellow eye of the same rabbit implanted with AGV without PMPC-coating. (TIF) [file pone.0252467.s001.tif]
